# ABO Blood Group and Endometrial Carcinoma: A Preliminary Single-Center Experience from Saudi Arabia

**DOI:** 10.7759/cureus.1959

**Published:** 2017-12-18

**Authors:** Ahmed Abu-Zaid, Mohannad Alsabban, Mohammed Abuzaid, Osama Alomar, Ismail A Al-Badawi, Hany Salem

**Affiliations:** 1 College of Graduate Health Sciences, University of Tennessee Health Science Center; 2 Obstetrics & Gynecology, King Faisal Specialist Hospital and Research Centre, Riyadh, Saudi Arabia; 3 Obstetrics & Gynecology, King Faisal Specialist Hospital and Research Center Riyadh, Saudi Arabia

**Keywords:** abo blood group, endometrial cancer, prognosis, saudi arabia

## Abstract

Introduction: Inherited ABO blood groups have been shown to play possible contributions in the pathogenesis of various gynecologic and non-gynecologic carcinomas. With regard to gynecologic carcinomas, there is a confined number of studies that explored the relationship between ABO blood group and endometrial carcinoma (EC) in the PubMed-indexed literature. To the best of our knowledge, no such study has ever been conducted in Saudi Arabia.

Objectives: Our study has two objectives: (I) to determine the prevalence of ABO blood groups among Saudi patients with EC, and (II) to explore the relationship between ABO blood group and several clinico-pathological prognostic parameters (namely: menopausal status [age], body mass index [BMI], tumor grade, FIGO [Fédération Internationale de Gynécologie et d'Obstétrique] stage and recurrence) in Saudi patients with EC.

Materials and Methods: A retrospective cross-sectional study from 01-January-2010 to 31-July-2014 was conducted at King Faisal Specialist Hospital & Research Centre, Riyadh, Saudi Arabia — a referral tertiary healthcare institute. One-hundred and fourteen patients (n=114) were included in the study. Clinico-pathological data were extrapolated from medical records, and their association with ABO blood groups were evaluated. Categorical data were presented as number of cases (n) and percentages (%). Two-tailed Chi-square test was used for univariate analysis. For all purposes, p values <0.05 were regarded as statistically significant.

Results: The mean age and BMI were 59.5 ± 10.8 years (range: 31 – 90) and 36.6 ± 8.6 kg/m^2^ (range: 17 – 60), respectively. The vast majority of patients were post-menopausal (86%), had BMI >28 kg/m^2 ^(84.2%), diagnosed with early FIGO stage I-II (76.3%) and developed no recurrence (86.8%). The frequencies of ABO blood group types A, B, AB, and O were 28.1%, 12.3%, 3.5% and 56.1%, respectively. When ABO blood groups were analyzed as four different types (A, B, AB and O), O-type was the most common ABO blood group in pre- and post-menopausal EC patients (43.8% and 58.2%, respectively; p=0.14). There were no statistically significant correlations between ABO blood groups and all the examined clinico-pathological factors. Moreover, when ABO blood groups were analyzed as two different types (O and non-O), similar results were obtained; no statistically significant correlations were found between ABO blood groups and all the examined clinico-pathological factors.

Conclusions: O-type was the most prevalent ABO blood group among Saudi Arabian patients with EC, and our finding was different from the existing literature, probably highlighting an ethnic-related variance. Furthermore, no statistically significant correlations were identified between ABO blood groups and all the examined clinico-pathological factors. Also, routine ABO blood group may emerge as a clinically accessible, beneficial and economical biomarker for a possible EC vulnerability. A large-sized case-control study is needed to withdraw solid conclusions.

## Introduction

Endometrial carcinoma (EC) is the most commonly diagnosed gynecologic neoplasm in developed countries, whereas it is the second most commonly diagnosed gynecologic neoplasm in developing countries [1]. Several risk factors have been demonstrated to play pivotal roles in the development of EC. Such important risk factors comprise: early menarche, late menopause, nulliparity, obesity, diabetes mellitus, old age, unopposed estrogen exposure, tamoxifen therapy, polycystic ovarian syndrome, and family history of breast, endometrial, ovarian or colon carcinoma [2-3].

Identification of genetic risk factors for EC continues to be an important area of research. Specifically, inherited ABO blood groups have been shown to play key contributions in the pathogenesis of various gynecologic and non-gynecologic carcinomas [4]. With regard to gynecologic carcinomas, there is a confined number of studies that explored the relationship between ABO blood group and EC in the PubMed-indexed literature [4-9]. Generally, these studies showed that ABO blood groups were present in characteristic proportions, as well as associated with distinctive clinico-pathological parameters and survival outcomes in patients with EC. However, to a certain degree, the results were inconsistent among the studies, and many failed to demonstarte statistically significant correlations. To the best of our knowledge, no such study has ever been conducted in Saudi Arabia, specifically, or from a developing third-world country, generally.

Thus, our retrospective study has two objectives: (I) to determine the prevalence of ABO blood groups among patients with EC, and (II) to explore the relationship between ABO blood group and several clinico-pathological prognostic parameters (namely: menopausal status [age], body mass index [BMI], tumor grade, FIGO [Fédération Internationale de Gynécologie et d'Obstétrique] stage and recurrence) in Saudi Arabian patients with EC.

## Materials and methods

The study took place at King Faisal Specialist Hospital & Research Centre (KFSH&RC), Riyadh, Saudi Arabia — a referral tertiary healthcare institute. The study protocol was approved by the Research Advisory Council (RAC) and Institutional Review Board (IRB) at KFSH&RC, Riyadh, Saudi Arabia.

From 01-January-2010 to 31-June-2014, all patients with pathologically confirmed diagnosis of EC were identified and retrospectively analyzed for clinico-pathological details (n=254).

Clinico-pathological details included: ABO blood group, age, menopausal status, body mass index (BMI), tumor grade, International Federation of Gynecology and Obstetrics (FIGO) stage, and recurrence. ABO blood group was determined by automated conventional hematology analyzers. The histological classification of EC was based on the World Health Organization classification of tumors [10]. Tumors were graded as follows: well (grade I), moderately (grade II) and poorly (grade III) differentiated tumors according to the FIGO grading system. FIGO stage was determined according to the revised 2009 FIGO staging system [11]. Recurrence was evaluated based on clinical, laboratory and imaging tests.

Exclusion criteria from study analysis included: missing ABO blood group, history of gynecologic or colon cancer, history of polycystic ovarian syndrome, concurrent tamoxifen therapy, endometrial thickness more than 15 mm while on hormonal replacement therapy, and follow-up period less than six months.

As per hospital’s protocol, all patients were followed up regularly at the outpatient clinic. The follow-up work-up included routine physical examination and vault smear. Chest X-ray, whole-body computed tomography (CT) scan, and position emission tomography/CT scan were done as seen clinically appropriate.

The categorical prognostic factors of menopausal status (pre-menopausal [age ≤49 years] vs. post-menopausal [age >49 years]), BMI (≤28 kg/m^2^ vs. >28 kg/m^2^), tumor grade (I vs. II-III), FIGO stage (I-II vs. III-IV) and recurrence (no vs. yes) were dichotomized into two groups. The age cut-off of menopause (that is, 49 years) was based on a recent study pertaining to the Saudi Arabian population [12]. The cut-off of BMI (≤28 vs. >28 kg/m^2^) was used in a previous EC related study [7]. FIGO stages I-II were referred to as early stages whereas FIGO stages III-IV were referred to as advanced stages [13]. Tumor grade I was referred to as a favorable grade whereas tumor grades II-III were referred to as unfavorable grades [13]. ABO blood groups were categorized into A, B, AB and O types. Further categorization of ABO blood group included O and non-O types.

Numerical data were presented as mean ± standard deviation [SD] (range: minimum – maximum). Categorical data were presented as number of cases (n) and percentages (%). Two-tailed chi-square test was used for univariate analysis. All statistical analyses were performed using IBM SPSS software version 22 for Windows (IBM Corp., Armonk, NY). For all purposes, p values <0.05 were regarded as statistically significant.

## Results

A total of a hundred and fourteen (n=114) patients met the study’s inclusion criteria and were included in the study analysis. The mean age and BMI were 59.5 ± 10.8 years (range: 31 – 90) and 36.6 ± 8.6 kg/m^2^ (range: 17 – 60), respectively. Around 83.2% (n=95), 45.6% (n=52) and 44.7% (n=51) of patients were parous, diabetic and hypertensive, respectively (data not shown). 

Table 1 shows the patient characteristics and univariate associations between ABO blood groups and clinico-pathological factors for EC. The vast majority of patients were post-menopausal (86%), had BMI >28 kg/m^2^ (84.2%), diagnosed with early FIGO stage I-II (76.3%) and developed no recurrence (86.8%). The frequencies of ABO blood group types A, B, AB, and O were 28.1%, 12.3%, 3.5%, and 56.1%, respectively. The frequencies of ABO blood group types non-O and O were 43.9% and 56.1%, respectively.

**Table 1:** Patient characteristics and univariate associations between ABO blood groups and several clinico-pathological factors for endometrial carcinoma NS: not significant ^1^ ABO blood groups were analyzed as four types (A, B, AB and O) ^2^ ABO blood groups were analyzed as two types (non-O and O) In ^1^ and ^2^, percentages (%) were calculated according to the total of individual row categories (that is, clinico-pathological factors) * Two-tailed chi-square test; statistical significance was determined as p<0.05

	All patients (total= 114) n (%)		ABO Blood Group^1^						ABO Blood Group^2^		
			A (n=32)	B (n=14)	AB (n=4)	O (n=64)	p value*		Non-O (n=50)	O (n=64)	p value*
Menopausal status											
Pre-menopause	16 (14)		4 (25)	3 (18.8)	2 (12.5)	7 (43.8)	NS		9 (56.3)	7 (43.8)	NS
Post-menopause	98 (86)		28 (28.6)	11 (11.2)	2 (2)	57 (58.2)			41 (41.8)	57 (58.2)	
Body mass index											
≤ 28 kg/m^2^	18 (15.8)		6 (33.3)	2 (11.1)	0 (0)	10 (55.6)	NS		8 (44.4)	10 (55.6)	NS
> 28 kg/m^2^	96 (84.2)		26 (27.1)	12 (12.5)	4 (4.2)	54 (56.3)			42 (43.8)	54 (56.3)	
FIGO stage											
I/II	87 (76.3)		22 (25.3)	11 (12.6)	4 (4.6)	50 (57.5)	NS		37 (42.5)	50 (57.5)	NS
III/IV	27 (23.7)		10 (37)	3 (11.1)	0 (0)	14 (51.9)			13 (48.1)	14 (51.9)	
Tumor Grade											
I	53 (46.5)		15 (28.3)	6 (11.3)	2 (3.8)	30 (56.6)	NS		23 (43.4)	30 (56.6)	NS
II/III	61 (53.5)		17 (27.9)	8 (13.1)	2 (3.3)	34 (55.7)			27 (44.3)	34 (55.7)	
Recurrence											
Yes	15 (13.2)		5 (33.3)	2 (13.3)	1 (6.7)	7 (46.7)	NS		8 (53.3)	7 (46.7)	NS
No	99 (86.8)		27 (27.3)	12 (12.1)	3 (3)	57 (57.6)			42 (42.4)	57 (57.6)	

When ABO blood groups were analyzed as four different types (A, B, AB and O), O-type was the most common ABO blood group in pre- and post-menopausal EC patients despite no statistical significance (43.8% and 58.2%, respectively; p=0.14). There were no statistically significant correlations between ABO blood groups and all the examined clinico-pathological factors. Moreover, when ABO blood groups were analyzed as two different types (non-O and O), similar results were obtained; no statistically significant correlations were found between ABO blood groups and all the examined clinico-pathological factors.

## Discussion

The ABO gene is located on chromosome number nine, and it has been shown that different ABO blood groups may influence various cancerous and non-cancerous related processes within the body [4]. Precise mechanisms underlying the role of ABO blood group in cancer pathogenesis are not well understood. However, several assumptions have been proposed. It could be possible that particular blood group antigens may aid neoplastic cells to undertake more aggressive biological behaviors [4]. Specifically, it has been demonstrated that existence of A antigen may boost cellular motility and smoothly expedite the cellular interactions between neoplastic cells [14]. Also, it has been noted that ABO blood group antigens may participate in conferring aggressive resistance to the immune system, generally, and programmed cell death (apoptosis), specifically [15]. Furthermore, a previous study revealed associations between ABO blood groups and altered levels of molecules implicated in inflammation, immune defense, cell adhesion and cellular signaling [16].

Pertaining to gynecologic malignancies, there are limited studies that explored the relationship between ABO blood group and EC [4-9]. In our study, overall, O-type was the most frequently implicated ABO blood group in patients with EC. Our finding mirrored a study conducted in Georgia [6]. However, our finding was in contrast to several other studies conducted elsewhere in Siberia [4], China [5] and Italy [7-8] — all of which showed that A-type was the most common ABO blood group present in patients with EC. Moreover, our study finding was in disagreement with an Armenian study which showed that AB-type was the most common ABO blood group in patients with EC [9].

To a larger degree, definitive correlations between particular ABO blood groups and risk of EC can be withdrawn from case-control studies. O-type was the most common ABO blood group in the control populations of China and Siberia. However, A-type ABO blood group was associated with a statistically significant increased risk of EC in the diseased Chinese population [5], and without a statistically significant increased risk of EC in the diseased Siberian population [4].

Collectively, our results suggest that O-type is the most prevalent ABO blood group among Saudi Arabian patients with EC. Moreover, it cannot be concluded that O-type ABO blood group may be associated with EC susceptibility. Bashawri and colleagues [17] reviewed the prevalence of ABO blood group in control obstetric Saudi populations across different cities, and found a high prevalence of O-type ABO blood group, ranging from 48% to 53%. These studies had small sample sizes ranging from as low as 150 to as high as 1,000 females per study, which are not sufficient enough to establish that O-type is the most prevalent ABO blood group in the Saudi Arabian obstetric population. This raises the possibility that, in our study, O-type ABO blood group may be associated with a possible EC susceptibility. However, a concrete conclusion cannot be withdrawn at this point and a nationwide large-sized case-control study is needed. More clearly, our finding highlights dissimilar results from literature which may be largely attributed to an ethnic-related difference.

In our study, O-type was the most common ABO blood group in pre- and post-menopausal EC patients (43.8% and 58.2%, respectively; p=0.14). In a dissimilarity to our finding, Nakashidze et al. [6] found that in Adjara population (Georgia), A-type and O-type were the most commonly implicated ABO blood groups in the pre- and post-menopausal EC patients, respectively.

Our findings showed that there were no statistically significant associations between ABO blood group and BMI, tumor grade and FIGO stage. This may be attributed to the small-sized sample size we had in our study. However, these findings were also echoed in a previous study [7].

Disease-free survival (DFS) and overall survival (OS) were not explored in our study. However, a previous study (Cox multivariate analysis) failed to observe a statistically significant association between ABO blood groups and DFS/OS in patients with EC [7]. Conversely, an Italian study demonstrated a reasonably improved five-year and 10-year survival in patients with O-type ABO blood group when compared to patients with A-type ABO blood group. Further research is needed to resolve the conflict.

Our study has several strengths. To the best of our knowledge, we report the first ever study from a developing country, generally, and Saudi Arabia, specifically, on the role of ABO blood group in EC. Largely, our results contribute substantial knowledge regionally, as well as internationally by enriching the very limited current body of literature [4-9]. A related systematic review with/without meta-analysis on the role of ABO blood group in EC is an interesting prospective research direction. Our future research includes a large-sized, multi-center, case-control study with an in-depth exploration of clinico-pathological factors and survival outcomes (DFS and OS) in patients with ovarian, endometrial and cervical cancers. Thus, more solid correlations and conclusions can be withdrawn.

Our study has several limitations that need to be addressed. Some of these limitations were acknowledged in the previous related studies [4-9]. Such limitations included the retrospective cross-sectional study design, and lack of a stronger design — that is, a case-control study. Potential confounders could not be excluded completely, for example, smoking status. In addition, this study originated from a single-center from Saudi Arabia, and hence its results may be ethnically-limited and without country-wide generalizability.

## Conclusions

In conclusion, O-type was the most prevalent ABO blood group among Saudi Arabian patients with EC, and our finding was different from the existing literature, probably highlighting an ethnic-related variance. Furthermore, no statistically significant correlations were identified between ABO blood groups and all the examined clinico-pathological factors. Also, routine ABO blood group may emerge as a clinically accessible, beneficial and economical biomarker for a possible EC vulnerability. A large-sized, multi-center, case-control study is needed to withdraw solid correlations and conclusions.
